# Study on an SIHRS Model of COVID-19 Pandemic With Impulse and Time Delay Under Media Coverage

**DOI:** 10.1109/ACCESS.2021.3064632

**Published:** 2021-03-09

**Authors:** Xinghua Chang, Jianrong Wang, Maoxing Liu, Zhen Jin, Dun Han

**Affiliations:** School of ScienceNorth University of China 66291 Taiyuan 030051 China; School of Mathematics ScienceShanxi University 12441 Taiyuan 030006 China; Complex Systems Research CenterShanxi University 12441 Taiyuan 030006 China; School of ScienceJiangsu University 12676 Zhenjiang 212013 China

**Keywords:** Media coverage, policies and regulations, COVID-19 pandemic, impulse and time delay, negative emotions

## Abstract

Media coverage plays an important role in prevention and control the spread of COVID-19 during the pandemic. In this paper, an SIHRS model of COVID-19 pandemic with impulse and time delay under media coverage is established. The positive and negative emotions of public are considered by the impact of confirmed cases and medical resources. In order to restrain the negative information of public, the factor of policies and regulations with impulse and time delay is introduced. Furthermore, the system model is simulated and verified by the reported data of COVID-19 pandemic in Wuhan. The main results are as follows: (1) When the implementation rate of the negative information generated by the confirmed cases gradually reduced to 0.4 times, the cumulative confirmed cases will be significantly reduced to about 37000, indicating that the popularization of pandemic related media information should be broad; (2) When the implementation rate affected by the amount of policies and regulations information gradually increases to 3 times, the cumulative confirmed cases will be significantly reduced to about 28000, indicating that the policies and regulations information should be continuously and incrementally reported; (3) When the inhibition rate of policies and regulation information on negative information gradually increases to 3 times, the cumulative confirmed cases will also be significantly reduced to about 27000 cases, indicating that the targeted policies and regulations information has a significant impact on inhibiting the corresponding negative emotions.

## I. Introduction

The outbreak of infectious diseases not only endangers people’s life and health, but also has a heavy blow on social order, economic development and social stability. Since December of 2019, the Corona Virus Disease 2019 (COVID-19) has broken out and continued to spread in Wuhan. The ways of the spread of COVID-19 pandemic mainly includes direct spread, aerosol spread and contact spread. The infection ability of COVID-19 is very strong and has a long incubation period, that leads to the rapid spread in an extremely covert form. Due to the peak period of the Spring Festival transportation in China, population flow and crowded gathering made the spread of COVID-19 pandemic faster and more widely. According to the transmission characteristics and spread pattern of COVID-19, the resumption time of Wuhan and the surrounding areas was analyzed based on the reported data [1]–[3]. A dynamic model of COVID-19 pandemic with time delay was presented to predict the trend of the pandemic situation [4]. The Susceptible-Exposed-Infectious-Recovered (SEIR) model represents a typical infectious epidemic disease using four distinctive phases, which is used for studying the spread of COVID-19 pandemic [5]–[7]. For the prediction of COVID-19 pandemic, some research results are based on the statistical analysis of data-driven [8]. The general public containment efforts or individual behavioral changes were considered to effectively remove individuals from the interaction dynamics or significantly reduce their participation in the transmission dynamics [9]. Due to the reduction of long-distance travel, the mobility network became more local and lattice-like during the COVID-19 pandemic in Germany [10]. Currently, the pandemic situation in some countries is spreading rapidly. The main reason is that there are less media coverage and weaker awareness of prevention and control.

The social impact of media coverage means the influence and effect of thoughts, feelings, attitudes or behaviors of public produced from some social information through media coverage. With the development of science and technology, public can know more about the latest epidemic information, control status and related knowledge through a variety of communication terminals. So that the public can enhance their self-care awareness and prevention ability. The impact of media coverage and health education on infectious diseases was described by affecting the infection rate of diseases directly. A large number of mathematical models with social impact in epidemiology have been studied, such as mass media coverage [11]–[16], consciousness and psychological effects [17]–[20].

Some studies have directly affected the impact of media coverage on infection rate of diseases to reduce the probability of infection. A three-dimensional compartments model was used to study the impact of media coverage on the spread and control of infectious diseases in specific areas (such as Severe Acute Respiratory Syndrome (SARS)) [11]. An Susceptible-Infectious-Susceptible (SIS) infection model was proposed to incorporate the media and education impact on the spread of the infectious disease in a given population [12]. In view of the dynamics of infectious disease transmission with isolation control strategy, an Susceptible-Exposed-Quarantined-Infectious-Hospitalized-Recovered-Susceptible (SEQIHRS) infectious disease model of the deterministic and nonlinear system was proposed [13]. Based on these studies, an SIS pandemic model on two kinds of plaque was established. In each region, media coverage of cases in local population could lead to a conscious behavior that one minimizes contact with the source of infection, thereby reducing the transmission rate of infection [14]. Furthermore, an SIS network model incorporating the influence of media coverage on transmission rate was formulated and analyzed on complex networks [15]. An SIS stochastic dynamics model with media coverage was proposed to study the impact of environmental fluctuations on disease dynamics [16].

Some scholars have committed to study the impact of awareness and psychology from the media coverage of infectious disease. Since the media coverage of the epidemic information could influence the psychology of public, a regional model was proposed to illustrate a possible mechanism for multiple outbreaks or even sustained periodic oscillations of emerging infectious diseases [17]. Considering the impact of local correlations, the model was introduced by a pair approximation in a well mixed-population [18]. A deterministic transmission and vaccination model was established to study the impact of media coverage on the dynamics of influenza transmission [19]. Based on media or psychological effects, a mathematical model with segmental smooth incidence rate of infectious disease was proposed and analyzed [20].

In some literatures, the media influence as an independent compartment was considered, and leads to the movement of the unaware to the aware population because of interaction. A non-linear mathematical model for the effects of awareness programs on the spread of infectious diseases such as flu has been proposed and analyzed [21]. Based on this, a non-linear mathematical model is proposed to assess the impact of creating awareness by the media on the spread of vector borne diseases [22]. Time delay is an important factor on the spread of infectious diseases from the intervention of media coverage. A mathematical model to study the impact of awareness programs on an infectious disease outbreak was proposed and analyzed [23].

There are also many studies on the influence of media coverage for the spread of infectious diseases during the actual epidemic period. Affected by the 2009 H1N1 (Hemagglutinin-1- Neuraminidase-1) influenza pandemic in South Korea, two different vector effects to modify the previous influenza transmission model have been proposed [24]. A binge drinking model with the impact of media in the scale free network was proposed [25]. A deterministic dynamical model to explore the interaction of the COVID-19 progression and the media coverage was proposed [26].

Thus it can be seen that media coverage was considered as a positive factor for most of research results. However, in reality, the influence of media coverage has two sides. The extreme and one-sided media coverage causes negative panic, which will lead to bad behaviors. So the comprehensive and detailed information from media coverage is required, and the collaborative integration of resources, laws, policies and other factors needs to be considered. Therefore, in view of the positive information, negative information and policy information generated by media during the COVID-19 pandemic, in this paper an SIHRS model of COVID-19 pandemic with impulse and delay under media coverage and policies and regulations will be established. The impact of negative emotions generated by the information of confirmed cases and medical resource will be considered. In order to restrain the negative information of the public, the publicity of policies and regulations with time delay and impulsive response will be proposed.

The rest of this paper are organized as follows. In section 2, an SIHRS model of COVID-19 pandemic with impulse and time delay under media coverage is proposed, and the related parameters are described in detail. In section 3, the numerical simulations are discussed with the reported data of Wuhan for the SIHRS model of COVID-19 pandemic. In section 4, the research results are summarized and discussed.

## II. Main Contents

**FIGURE 1.**
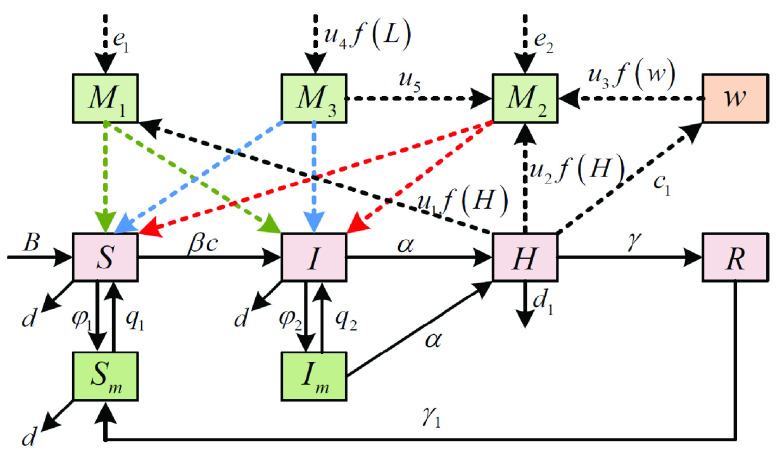
The state transformation diagram of SIHRS model with multi-dimensional information under media coverage.

Usually, media coverage plays an immeasurable role in the prevention and control of infectious diseases. There are many research on the positive emotion which is generated by media coverage. Yet, the negative emotion, is also generated by media coverage, which leads to some extreme behaviors. In order to restrain the negative information of public, the information of policies and regulations is propagated by media coverage. In addition, the information generated by media coverage has obvious impulse characteristic and time-delay characteristic. Therefore, according to epidemiology regularity, intervention measures and media coverage of COVID-19 pandemic, an SIHRS pandemic model with impulse and time delay will be established. During the pandemic period, the information generated from media coverage is divided into three categories: positive information, negative information and policies and regulations information. We divide the whole population into the following six categories: $S(t)$, $S_{m}(t)$, $I(t)$, $I_{m}(t)$, $H(t)$ and $R(t)$. $S(t)$ is the susceptible without active isolation and protection awareness. $S_{m}(t)$ is the susceptible with active isolation and protection awareness. $I(t)$ refers to asymptomatic infection and symptomatic unconfirmed infection who has infectious capacity but no isolation treatment. $I_{m}(t)$ indicates that the patient has the awareness to do self-protection or self-isolation under the influence of media information but no isolation treatment. $H(t)$ means that the patients who have been confirmed by clinical diagnosis or screening, and hospitalized for isolation treatment. $R(t)$ denotes that the cured person who have immunity after medical treatment, but will be a susceptible $S_{m}(t)$ with immune failure after a period of time. Then $S(t)+S_{m}(t)+I(t)+I_{m}(t)+H(t)+R(t)=N(t)$. In addition, we will also consider the impact of medical resources, positive information, negative information, policies and regulations information on the prevention and control of COVID-19 pandemic. $w(t)$ represents medical resources in a certain area, and includes medical equipment, beds, medical staff, etc. $M_{1}(t)$ means that the effective information of positive emotions, which is caused by the pandemic information through the media coverage. Analogously, $M_{2}(t)$ means that the effective information of negative emotions. $M_{3}(t)$ means that the effective information is generated from policies and regulations of pandemic situation, and which can restrain the negative emotions of the public. Based on the above description, the state transition diagram of SIHRS model with the impact of multi-dimensional information under media coverage is shown as Figure 1.

Next, the SIHRS model of pandemic dynamic system is given:

\begin{align*} \begin{cases} \dfrac {dS(t)}{dt}=B-\beta c S(t)(I(t)+\theta H(t))-\varphi _{1} S(t)Y(t)\\ \qquad \qquad ~\,\,+q_{1}S_{m}(t)-dS(t),\\ \dfrac {dS_{m}(t)}{dt}=\varphi _{1} S(t)Y(t)-q_{1}S_{m}(t)+\gamma _{1}R(t)-dS_{m}(t),\\ \dfrac {dI(t)}{dt}=\beta c S(t)(I(t)+\theta H(t))-\varphi _{2} I(t)Y(t)-\alpha I(t)\\ \qquad \qquad \,\,+q_{2}I_{m}(t)-dI(t),\\ \dfrac {dI_{m}(t)}{dt}=\varphi _{2} I(t)Y(t)-\alpha I_{m}(t)-q_{2}I_{m}(t),\\ \dfrac {dH(t)}{dt}=\alpha I(t)+\alpha I_{m}(t)-\gamma H(t)-d_{1}H(t),\\ \dfrac {dR(t)}{dt}=\gamma H(t)-\gamma _{1}R(t),\\ \dfrac {dw(t)}{dt}=r\dfrac {w(t)(W-w(t))}{W}-c_{1}H(t),\\ \dfrac {dM_{1}(t)}{dt}=e_{1}+u_{1}f(H(t))-\mu _{0}M_{1}(t),\\ \dfrac {dM_{2}(t)}{dt}=e_{2}+u_{2}f(H(t))+u_{3}f(w(t))-u_{5}M_{3}(t)\\ \qquad \qquad \quad \,\,-\mu _{0}M_{2}(t),\\ \dfrac {dM_{3}(t)}{dt}=u_{4}f(L)-\mu _{0}M_{3}(t),\\ \end{cases}\tag{2.1}\end{align*}

where, the description, value and source of parameters are shown in Table 1. $Y(t)$ is a function which is the impact of $M_{1}+M_{3}$ reflected by the half-saturation constant $K$ [31], namely $Y(t)=\frac {M_{1}(t)-M_{2}(t)+M_{3}(t)}{K+M_{1}(t)+M_{3}(t)}$. $r\frac {w(t)(W-w(t))}{W}$ is the growth function of medical resources with Logistic mapping.

**TABLE 1:** The Description, Value and Source of Parameters

Parameters	Description	Value	Source
$B$	Population migration, mainly consider the medical staff and social volunteers.	2216 persons	[27]
$d$	Emigration rate of population.	0.0001 day^−1^	[27]
$\beta $	The probability of transmission per contact.	$2.0389\times 10^{-9}$ person $^{-1} \cdot $ day^−1^	[1]
$\theta $	The probability of transmission per contact during isolation treatment period.	0.022 person $^{-1} \cdot $ day^−1^	[28]
$\varphi _{1}$	The probability of a susceptible person consciously performing active protection or isolation through media information.	0.8 person $^{-1} \cdot $ day^−1^	Set
$\varphi _{2}$	The probability of consciously carrying out active protection and isolation through media information during incubation period.	0.85 person $^{-1} \cdot $ day^−1^	Set
$K$	The half-saturation constant of media information.	320 bits	Estimated
$q_{1}$	Conversion rate from $S_{m}$ to $S$.	0.05 day^−1^	Estimated
$q_{2}$	Conversion rate from $I_{m}$ to $I$.	0.03 day^−1^	Estimated
$q_{3}$	When a pulse occurs, conversion rate from $I_{m}$ to $I$.	0.01 day^−1^	Estimated
$\gamma _{1}$	Immune failure rate.	0.02 day^−1^	Set
$c_{1}$	The consumption rate of medical resources.	0.95 day^−1^	[29]
$r$	The intrinsic growth rate of medical resources.	0.13 day^−1^	Estimated
$W$	The total amount of medical resources in a certain area.	$2.6\times 10^{4}$ beds	[30]
$e_{1}$	The amount of positive information produced by other factors except confirmed case factor.	6 bit $\cdot $day^−1^	Set
$e_{2}$	The amount of negative information produced by other factors except confirmed case and medical resources factors.	2 bit $\cdot $day^−1^	Set
$u_{1}$	The implementation rate of positive information influenced by confirmed cases.	0.7 bit $\cdot $person $^{-1} \cdot $ day^−1^	Set
$u_{2}$	The implementation rate of negative information influenced by confirmed cases, and $u_{1}+u_{2}=1$.	0.3 bit $\cdot $person $^{-1} \cdot $ day^−1^	Set
$u_{3}$	The implementation rate of negative information influenced by medical resources.	0.01 bit $\cdot $bed $^{-1} \cdot $ day^−1^	Estimated
$u_{4}$	The implementation rate affected by the amount of policies and regulations information.	0.02 bit $\cdot $person $^{-1} \cdot $ day^−1^	Estimated
$u_{5}$	The inhibition rate of policies and regulations information to negative information.	0.15 bit $\cdot $day^−1^	Estimated
$\mu _{0}$	The dissipation rate of media information.	0.1 bit $\cdot $day^−1^	Estimated
$L$	The number of audiences who can be affected by policies and regulations information reports, the audience is the number of permanent residents in Wuhan.	$1.108\times 10^{7}$ persons	Set
$\delta $	The ratio of negative information to positive information.	0.2	Estimated
$\tau $	The time delay caused by the implementation of impulsive policies and regulations information.	5 days	[1]

In addition, the values of death rate, confirmed rate and recovery rate are different at different time stages. Therefore, the piecewise functions of their three factors are given, as shown in Table 2.

**TABLE 2:** The Description, Function and Source of Parameters

Parameter	Description		Function	Source
$d_{1}$	Death rate due to illness	In early stage (before February 17), the treatment efficiency was inefficiency, but in middle stage (February 17 to February 25), the number of confirmed cases increased significantly.	$0.009-0.000222t$ day ^−1^, $0 < t < 30$	Estimated
		The efficiency of treatment has been greatly improved.	0.008 day^−1^, $t\geq 30$	Estimated
$\alpha $	Confirmed rate from $I_{m}$ and $I$ to $H$	Nucleic acid detection.	0.19 day^−1^, $t < 17$	Estimated
		Nucleic acid detection and clinical diagnosis.	0.25 day^−1^, $t\geq 17$	Estimated
$\gamma $	Recovery rate of confirmed cases	In early stage, the level of medical treatment was low.	$0.002t$ day^−1^, $t < 17$	[29]
		In the middle stage, the level of medical treatment has been greatly improved.	$0.004t$ day^−1^, $17\leq t\leq 60$	Estimated
		In later stage, the recovery rate is stable.	0.24 day^−1^, $t\geq 60$	Estimated

$f(x)$ refers to the amount of information generated by media coverage on variable $x$. The amount of positive information, negative information, policies and regulations information are closely related to the number of people. For example, one hospital bed corresponds to one patient in medical resources, policies and regulations have significant impact on awareness of prevention and control of citizens in the region. Therefore, we assume that each person is one source symbol, and the probability of each source symbol is the same. Then according to Shannon theorem of information theory [32], we can know that the amount of information influenced by the number of confirmed cases is as follows:

\begin{align*}f(H(t))=\begin{cases} \log _{2}H(t),& H(t)\geq 1,\\ 0,& H(t)= 0.\\ \end{cases}\end{align*}

The amount of negative information produced by the impact of medical resources is

\begin{align*} f(w(t))=\begin{cases} e^{-\log _{2}w(t)}, & w(t)\geq 1,\\ 1,& -1\leq w(t)\leq 1,\\ e^{\log _{2}|w(t)|}, & w(t) < -1,\\ \end{cases}\end{align*}

where $w(t)\in Z$.

The amount of information generated by the impact of policies and regulations is $f(L)=\log _{2}L$, $L$ is the total population in the region.

The dynamics of system (2.1) is investigated in the following bounded region as follows:

\begin{align*} \Gamma \triangleq \left \{{ \begin{array}{llllllllll} (S, S_{m}, I, I_{m}, H, R, w, M_{1}, M_{2}, M_{3})\in \Re ^{10}:\\ \dfrac {B}{d+d_{1}}\leq S+S_{m}+I+I_{m}+H+R=N \leq \dfrac {B}{d},\\ -\dfrac {c_{1}B}{rd} \leq w\leq W, 0\leq M_{1}\leq \dfrac {e_{1}+u_{1}f\left ({\dfrac {B}{d}}\right)}{\mu _{0}},\\ 0\leq M_{2}\leq \dfrac {e_{2}}{\mu _{0}}+\dfrac {u_{2}}{\mu _{0}}f\left ({\dfrac {B}{d}}\right)+ \dfrac {u_{3}}{\mu _{0}}e^{f\left ({\dfrac {c_{1}B}{rd}}\right)},\\ \dfrac {u_{4}f(L)-1}{\mu _{0}}\leq M_{3}\leq \dfrac {u_{4}f(L)}{\mu _{0}}\\ \end{array} }\right).\end{align*}

According to the system model (2.1), the basic reproduction number $R_{0}$ is calculated by the next generation matrix method [33] as follows

\begin{equation*} R_{0}=\frac {B\beta c(\gamma +d_{1}+\alpha \theta)}{d(\alpha +d)(\gamma +d_{1})}.\tag{2.2}\end{equation*}

According to the system model (2.1), the disease-free equilibrium $E_{0}$ can be calculated as follows:

\begin{equation*}E_{0}=(S^{*}, S_{m}^{*}, 0, 0, 0, 0, W, M_{1}^{*}, M_{2}^{*}, M_{3}^{*}),\end{equation*}

where,

\begin{align*} S^{*}=&\frac {B(q_{1}+d)}{d(\varphi _{1}Y^{*}+q_{1}+d)}, \quad Y^{*}=\frac {M^{*}_{1}-M^{*}_{2}+M^{*}_{3}}{K+M^{*}_{1}+M^{*}_{3}}, \\ S^{*}_{m}=&\frac {B\varphi _{1}Y^{*}}{d(\varphi _{1}Y^{*}+q_{1}+d}, \\ M_{1}^{*}=&\frac {e_{1}}{\mu _{0}}, \\ M_{2}^{*}=&\frac {e_{2}+u_{3}f(W)}{\mu _{0}}-\frac {u_{4}u_{5}f(L)}{\mu _{0}^{2}}, \\ M_{3}^{*}=&\frac {u_{4}f(L)}{\mu _{0}}.\end{align*}

Due to $M_{1}^{*}-M_{2}^{*}+M^{*}_{3}\geq 0$, then $Y^{*}\geq 0$. From the values of relevant parameters in Table 1, it can be concluded that $M_{2}^{*}\geq 0$.

During the pandemic, when the negative emotion generated by media coverage reaches a certain degree, it will cause some sudden bad behaviors (such as panic, concealment, escape, looting, doing evil, etc.). Then, it may lead to further spread and outbreak of the disease, which is an obvious burden for the pandemic prevention work. Therefore, we use the impulse and delay theory to characterize the dynamic behavior affected by media coverage. Next, two pulse conditions are introduced into the model as:

1) If $M_{2}(t)>M_{3}(t)$, it is used to describe the negative information that cannot be offset by the weak awareness of policies and regulations.
2) If $M_{2}(t)>\delta M_{1}(t)$, when the ratio of negative information to positive information is greater than $\delta $, the pulse system will be triggered.

Based on the system model (2.1), a system model based on state pulse is proposed as follows:

\begin{align*} \begin{cases} S(t^{+})=S(t)-1.5 \beta c S(t)(I(t)+\theta H(t)),\\ S_{m}(t^{+})=S_{m}(t),\\ I(t^{+})=I(t)+1.5\beta c S(t)(I(t)+\theta H(t))+q_{3}I_{m}(t),\\ I_{m}(t^{+})=I_{m}(t)-q_{3}I_{m}(t),\\ H(t^{+})=H(t),\\ R(t^{+})=R(t),\\ w(t^{+})=w(t),\\ M_{1}(t^{+})=M_{1}(t),\\ M_{2}(t^{+})=M_{2}(t),\\ M_{3}((t+\tau)^{+})=M_{3}(t)+5u_{4}f(L).\\ \end{cases}\tag{2.3}\end{align*}

When the negative emotions increase, it will trigger pulse conditions and increase the contact rate which is set as $1.5c$. Meanwhile, in order to restrain the negative emotions, the information of policies and regulations is released. Then the implementation rate of policies and regulations information is set as $5u_{4}$. $M_{3}((t+\tau)^{+})$ means that the amount of information of policies and regulations is increased because of pulse after time delay $\tau $. Let $M(t)$ is the effective amount of information about the impact of media information on COVID-19 pandemic prevention and control at any time in a certain region, i.e. $M(t)=M_{1}(t)-M_{2}(t)+M_{3}(t)$.

## III. Numerical Simulation

### A. Data

From January 23, 2020, Wuhan was closed down. We mainly consider the number of hospital beds (from January 26 to February 25), cumulative confirmed cases (January 26 to February 13), recovered (January 26 to March 13) and deaths due to illness (January 26 to March 13) [27]–[30]. The permanent population of Wuhan is $N_{\textrm {Wuhan}}=1.108\times 10^{7}$.

### B. Initial Value Setting

The types, values and sources of the initial values in the system model (2.1) are given in Table 3.

**TABLE 3:** The Types, Values and Sources of the Initial Values

Type	Value	Source	Type	Value	Source
$S(0)$	1101342 persons	Set	$R(0)$	45 persons	[27]
$S_{m}(0)$	9972000 persons	Set	$w(0)$	4000 beds	[27]
$I(0)$	1034 persons	[27]	$M_{1}(0)$	7.125 bits	Estimated
$I_{m}(0)$	4034 persons	[27]	$M_{2}(0)$	3.112 bits	Estimated
$H(0)$	1460 persons	[27]	$M_{3}(0)$	1.134 bits	Estimated

### C. Data Fitting

The pandemic data of Wuhan was used for fitting the model. Since January 23, 2020, Wuhan had entered a state of emergency. Various media were competing to publicize and report the relevant pandemic information. The isolation and control measures were gradually strengthened, and the contact rate of the population was rapidly reduced. Considering that the contact rate $c$ is closely related to the amount of media information, the function of contact rate $c$ is given as follows:

\begin{align*} c(M)=\begin{cases} 10-\log _{2}M, &M\geq 1,\\ 10,& 0\leq M\leq 1.\\ \end{cases}\end{align*}

**FIGURE 2.**
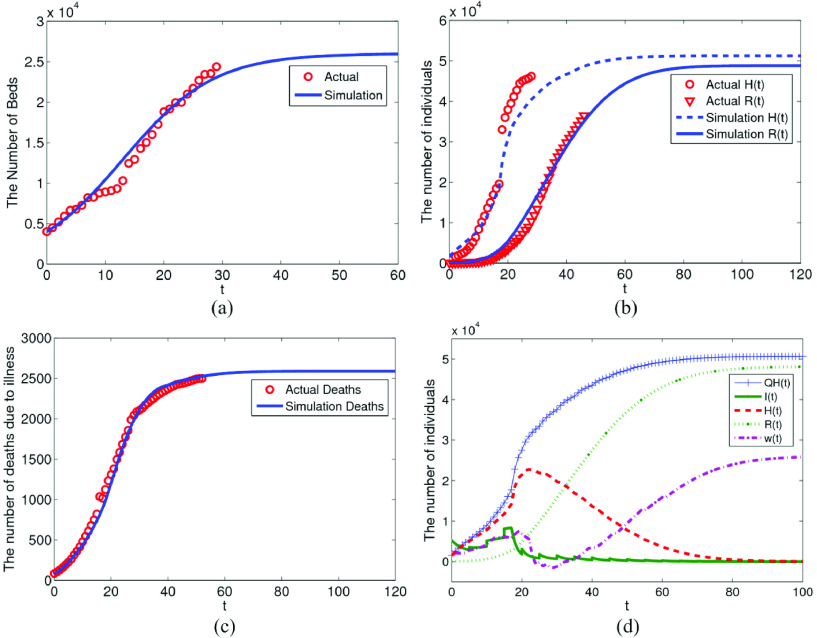
The fitting effect of sickbeds, cumulative confirmed cases, healers and deaths due to diseases.

From the initial values $M_{1}(0)$, $M_{2}(0)$, $M_{3}(0)$, through numerical calculation, we get $c(0)\approx 8$. The system model was fitted by using the data of hospital bed, cumulative confirmed cases, cured persons and deaths due to illness. The fitting effect is shown in Figure 2.

In Figure 2(a), logistic growth model was used to fit the bed data of major hospitals in Wuhan, and the total number of beds was 25594. In view of the change of diagnosis index on February 12, which further shortened the incubation period, a piecewise function was defined. However, in order to avoid data over fitting, it is necessary to consider the actual value when defining $\alpha $. So the sudden increase of confirmed cases is scattered into the number of new confirmed cases in the coming days. Therefore, the fitting effect of existing confirmed cases in Wuhan does not reach the actual peak. It can be seen from Figure 2(b) that the cumulative number of confirmed cases increased abruptly from February 13 to February 15. The fitting effect of death data due to illness is shown in Figure 2(c) using the data of deaths in Wuhan. In Figure 2(d), the cumulative of confirmed cases $QH$, latent period cases $I+I_{m}$, confirmed cases $H$, recovered $R$ and medical resources $w$ in the model system are fitted. It can be seen that due to the substantial increase of confirmed cases from February 13 to February 15, the shortage of medical resources occurred from February 17 to February 20.

**FIGURE 3.**
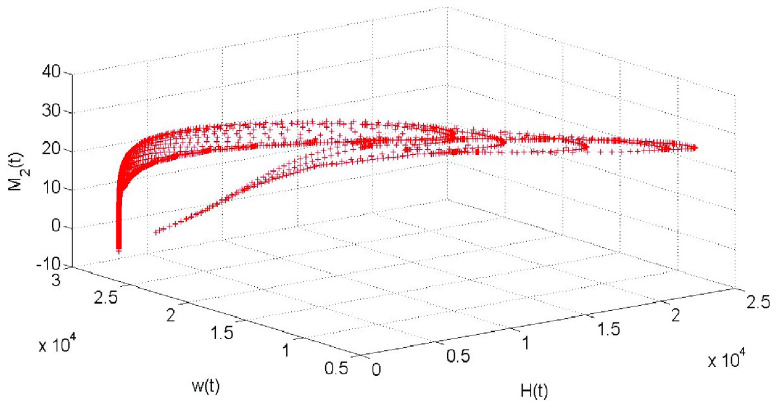
The influence of confirmed cases and medical resources on negative information.

The impact of confirmed cases and medical resources on the amount of negative information is shown in Figure 3. As can be seen, the shortage of medical resources is also increasing. In the middle and late stage of pandemic prevention and control, the number of confirmed cases has been greatly reduced with the continuous improvement of medical treatment efficiency. So medical resources have been largely idled, and then the amount of negative information has also disappeared.

**FIGURE 4.**
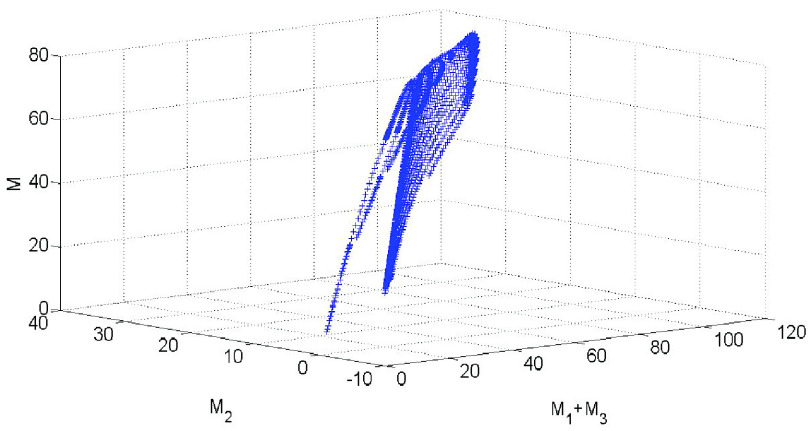
The influence of positive information, policies and regulations information and negative information on effective information.

During the whole pandemic period, the three kinds of information coexist and restrict each other. The positive information plays a positive role in promoting the prevention and control of the pandemic, while the negative information has a certain burden on the prevention and control of the pandemic. The amount of positive information and negative information increases rapidly in the early stage of the pandemic, but the pulse of policies and regulations information is at a low level. Therefore, the pulse condition will be triggered many times, which makes the amount of policies and regulations information increases to offset the negative information. The influence of positive information, policies and regulations information and negative information on effective information is shown in Figure 4. Combined with Figure 2, we can see that the amount of positive information and negative information is increased in the early stage. The effective information $M$ reaches the peak value 85 on the 29th day, and the minimum contact rate is calculated as $c=3.6$. In the middle and late stages of pandemic prevention and control, the amount of positive and negative information gradually decreases due to the effective treatment, compulsory isolation and legal policy publicity.

**FIGURE 5.**
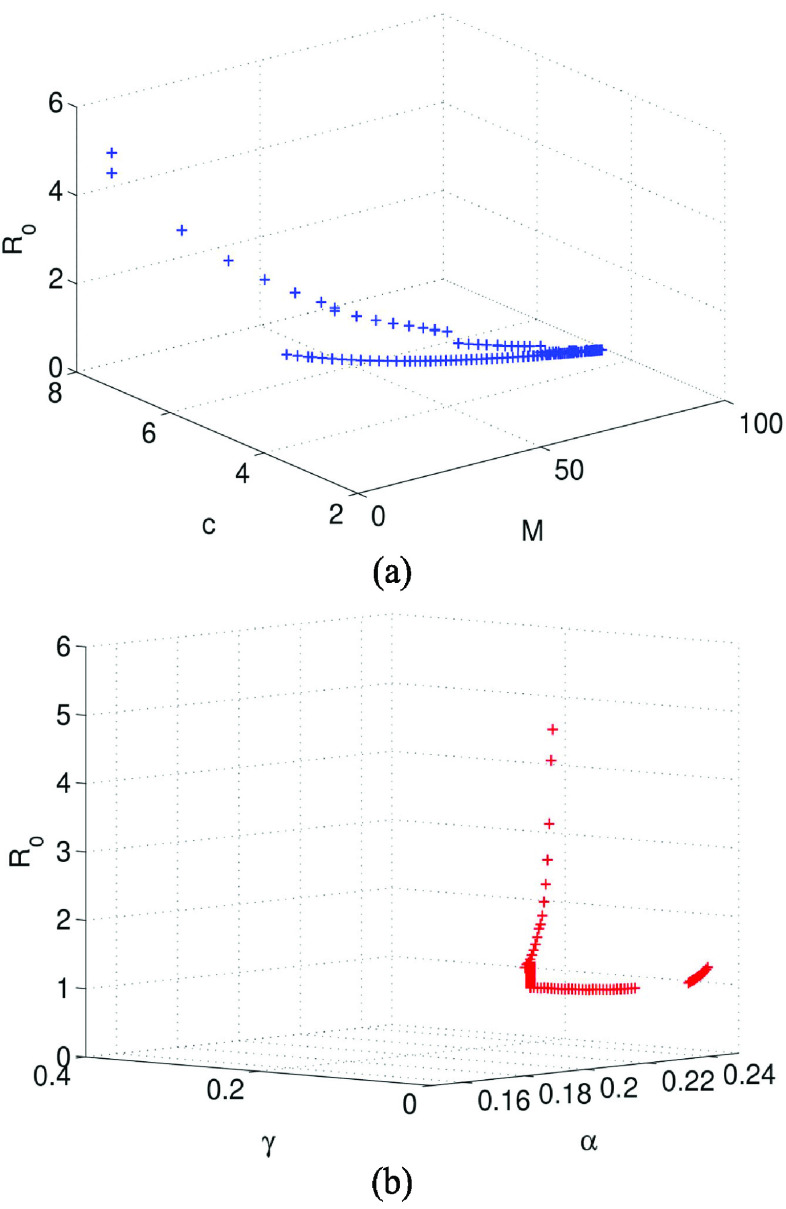
The influence on basic reproduction number $R_{0}$ from $c$, $M$, $\alpha $ and $\gamma $.

The impact of parameters $c$, $M$, $\alpha $, $\gamma $ on basic reproduction number is shown in Figure 5. It can be seen from Figure 5(a) that due to the increase of effective information, the contact rate gradually decreases in the early stage of the pandemic, and the basic reproduction number decreases rapidly from the maximum value 4.823 to less than 1 within 12 days. Due to the effective prevention and control of the pandemic, the amount of effective information gradually decreased in the later stage, which made the contact rate $c$ increased. So that the trend of $R_{0}$ will be greater than 1 after 120 days. Therefore, in order to prevent the second outbreak of the pandemic, we need to continue to do a good job in safety protection and policies and regulations information publicity. It can be seen from Figure 5(b) that the basic reproduction number $R_{0}$ decreases rapidly from the maximum value 4.823 to less than 1 with the gradual decreasing $\alpha $ and increasing $\gamma $.

**FIGURE 6.**
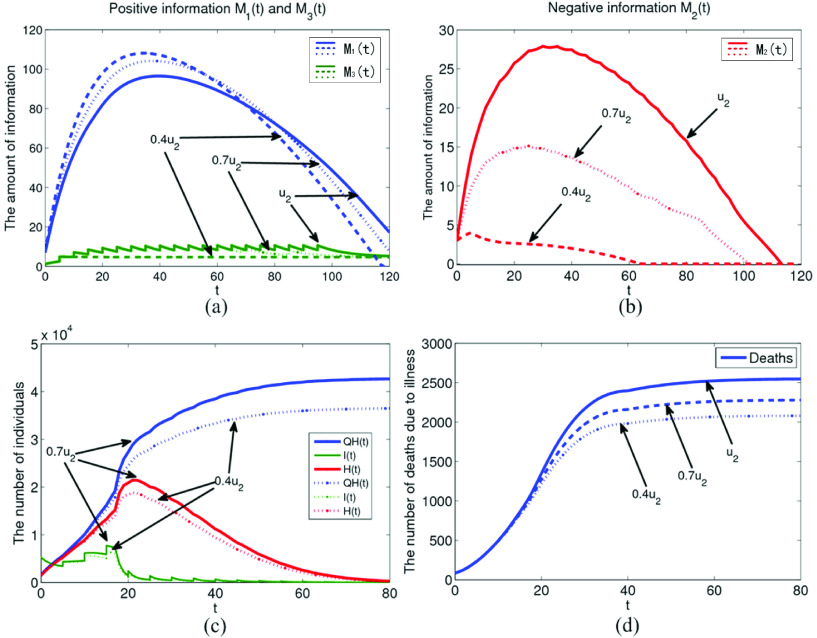
The influence of negative information generated by confirmed cases under implementation rate $u_{2}$ on system model.

Diversified media coverage for confirmed cases can enhance the public awareness about pandemic, and improve the public awareness of prevention and control. However, in practice, some people know little about the related information reports, so that negative emotions are formed easily leading to some extreme behaviors, such as escaping, cover-up and looting. In Figure 6, the effect of negative information, is generated by confirmed cases on the system model under the implementation rate $u_{2}$. Due to $u_{1}+u_{2}=1$, the value of $u_{2}$ are set to $u_{2}$, $0.7u_{2}$ and $0.4u_{2}$ in Figure 6, respectively. As $u_{2}$ gradually decreases, the amount of positive information $M_{1}$ increases significantly in the early and middle periods of pandemic prevention and control. In addition, in Figure 6(b) the amount of policies and regulations information generated by the pulse also decreases gradually. The main reason is that with the gradual decrease of $u_{2}$, the amount of negative information $M_{2}$ also decreases significantly. As can be seen from Figures 6(c) and 6(d), when $u_{2}$ is reduced to $0.4u_{2}$, the cumulative number of confirmed cases will be reduced to about 37000, and the number of deaths due to illness will also be reduced to about 2100. It can be seen that the media should fully consider the different groups when reporting the pandemic information. We should make good use of various media resources and spread the pandemic information to everyone.

**FIGURE 7.**
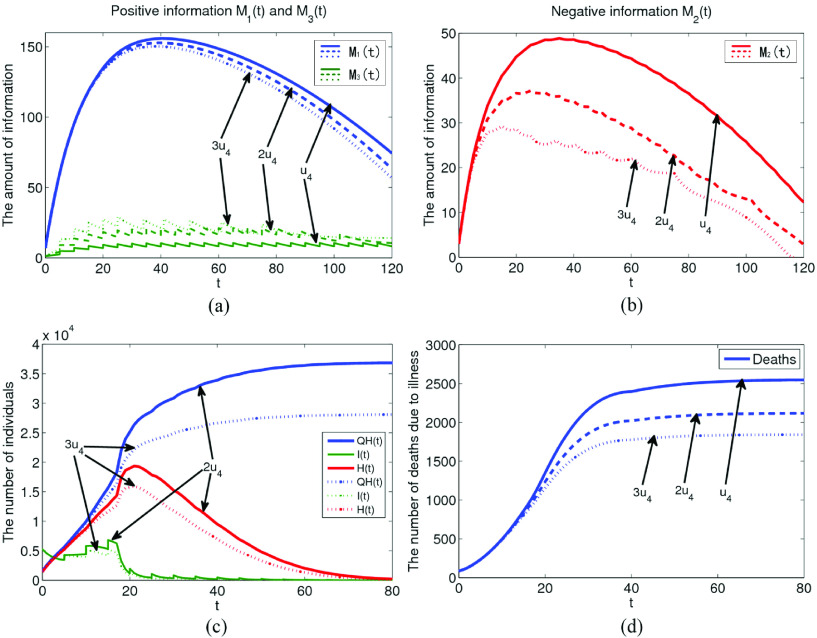
The influence of the information generated by policies and regulations under implementation rate $u_{4}$ on system model.

During the pandemic period, in order to achieve a certain purposes, some media or individuals spread all kinds of negative information (such as rumors, one-sided topics, etc.) which will seriously affect the public sentiment. Therefore, in order to maintain social order and stabilize public sentiment, it is very important to report and guide the information of policies and regulations. The effect of the amount of information generated by policies and regulations under the effect of implementation rate $u_{4}$ is shown in Figure 7. The values of $u_{4}$ are set to $u_{4}$, $2u_{4}$, $3u_{4}$ in Figure 7, respectively. With the gradual increase of $u_{4}$, the amount of policies and regulations information gradually increases, resulting in a significant decrease in the amount of negative information, as shown in Figure 7(b). As can be seen from Figures 7(c) and 7(d), when $u_{4}$ increases to $3u_{4}$, the total number of confirmed cases will be greatly reduced to about 28000, and the number of deaths due to illness will also be reduced to about 1700. It can be seen that the implementation of policies and regulations directly affects the negative emotions during the pandemic period. Therefore, it is necessary to continuously, deeply and widely report the policies and regulations related to the pandemic through various media to resist and guide the negative emotions of some people.

**FIGURE 8.**
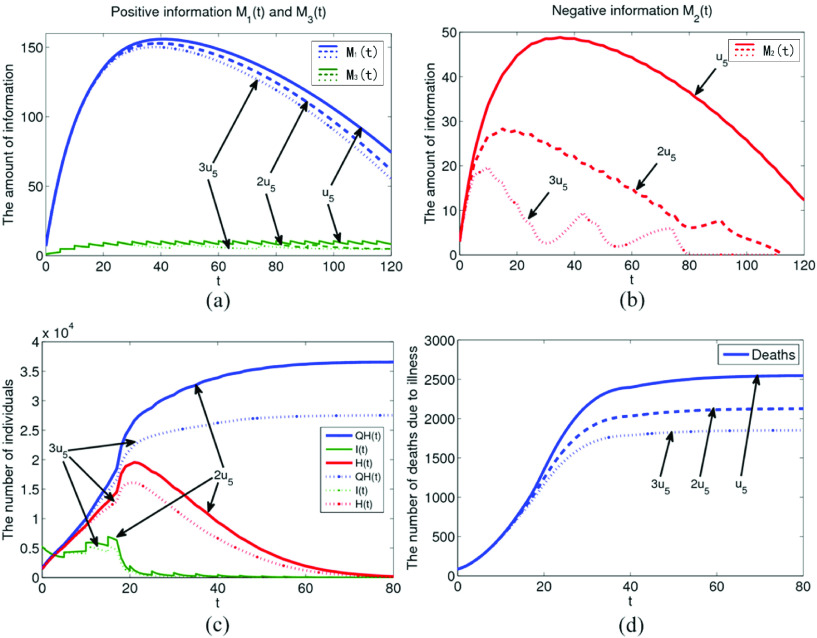
The influence of policies and regulations information on negative information under the inhibition rate $u_{5}$.

During the pandemic period, it is very important to report and guide the information of policies and regulations. However, it is particularly important to make corresponding policies and regulations for all kinds of negative emotions. The influence of information generated by policies and regulations on the negative information under the suppression rate $u_{5}$ is shown in Figure 8. The values of $u_{5}$ are set to $u_{5}$, $2u_{5}$, $3u_{5}$ in Figure 8, respectively. With the gradual increase of $u_{5}$, the amount of negative information $M_{2}$ decreases greatly, and the amount of policies and regulations information also gradually decreases, as shown in Figure 8(a). As can be seen from Figures 8(c) and 8(d), when $u_{5}$ increases to $3u_{5}$, the cumulative number of confirmed cases will also be greatly reduced to about 27000, and the number of deaths due to illness will also be reduced to about 1700. Therefore, targeted policies and regulations information has a good effect on suppressing the corresponding negative emotions. So it is necessary to analyze all kinds of negative emotions, understand the essential driving force of negative emotions, and formulate corresponding policies and regulations to restrain and guide public awareness.

## IV. Limitations of the Study

In this paper, although the research is innovative, it mainly predicts the trend of the popularity of COVID-19 pandemic from the perspective of engineering simulation analysis. Because of the complexity of the system, it is difficult to prove the stability of the system from the perspective of theoretical analysis. In the future research work, we will simplify the model on the basis of retaining the main innovation of the model, and make a detailed theoretical analysis. The research results will have universal guiding significance for some infectious diseases.

## V. Conclusion and Discussion

Aiming at the media information coverage influenced by multiple factors during the COVID-19 pandemic, in this paper an SIHRS model with impulse and time delay under the media coverage has been established. The positive and negative emotions of public are considered by the impact of confirmed cases and medical resources. In order to restrain the negative information of public, the factor of policies and regulations with impulse and time delay is introduced. Since the contact rate of the public is closely related to the effective amount of information reported by the media, the inverse proportional function between the close contact rate $c$ and the amount of information $M$ was given. Then, the data of COVID-19 pandemic in Wuhan from January 26 to February 25, 2020 was used for fitting the proposed system model. The basic reproduction number $R_{0}$ is calculated from the system equation (2.1), and the relationship between $R_{0}$ and the parameters was analyzed. It was shown that $R_{0}$ rapidly decreased to below 1 in the short term, but displayed a definite trend $R_{0}>1$ in the later stage. Therefore, in order to prevent the second outbreak of the pandemic, we need to continue to do a good job in safety protection and legal policy information publicity.

Then, qualitative and quantitative analysis were presented about three key parameters $u_{2}, u_{4}, u_{5}$, that affected the amount of negative information and took different values for each parameter to analyze its influence on the system model. With the gradual decrease of $u_{2}$ and the gradual increase of $u_{1}$, the amount of positive information $M_{1}$ increased significantly in the early and middle stages of pandemic prevention and control, which could effectively reduce the number of confirmed cases. It shows that the media should fully consider the characteristics of various groups of people, make effective use of various media resources to popularize the related knowledge of infectious diseases to everyone deeply and widely. With the gradual increase of $u_{4}$, the amount of policies and regulations information gradually increased, which makes the negative information $M_{2}$ decreased significantly, and also could effectively reduce the number of confirmed cases. Therefore, the implementation of policies and regulations information directly affected the negative emotions during the pandemic period. So it is necessary to continuously, deeply and extensively report the policies and regulations related to the pandemic situation through various media to suppress and guide the negative emotions of the people. With the gradual increase of $u_{5}$, the amount of negative information $M_{2}$ decreased significantly, which could also effectively reduce the number of confirmed cases. It shows that targeted policies and regulations information has a good effect on suppressing the corresponding negative emotions. So it is necessary to analyze the internal driving force of all kinds of negative emotions, and then managers should formulate corresponding policies and regulations to restrain and guide negative emotions. The results of this study can provide a certain reference for the media coverage during the pandemic period.

A large number of achievements focus on the impact of media information on the spread and control of infectious diseases. It is not hard to find out that the media coverage was considered as a positive factor. For example, a deterministic dynamical model was proposed to examine the interaction of the disease progression and the media reports and to investigate the effectiveness of media reporting on mitigating the spread of COVID-19 [26]. By taking the weight value of the number of media reports, the quantization of awareness information was realized. Sensitivity analysis suggested that, during the early phase of the COVID-19 outbreak, enhancing the response rate of the media reporting to the severity of COVID-19, and enhancing the response rate of the public awareness to the media reports, both could bring forward the peak time and reduce the peak size of the infection significantly. However, public awareness of disease was not directly proportional to the number of media coverage, but closely related to the content of media coverage (such as the number of confirmed cases, the number of beds, etc.). So, an SIHRS epidemic model with media coverage for COVID-19 pandemic was proposed [34]. The number of confirmed cases was used to quantify awareness information. It was shown that with the decrease of information implementation rate, the peak of confirmed cases would be delayed to reach, and would increase significantly. However, in reality, the public’s awareness information was affect by media coverage, including not only positive information, but also negative information, as well as the information of policies and regulations to suppress negative information. Therefore, the main innovations of this paper are as follows: (1) The influence of the coexistence of positive and negative information from media coverage on the spread and control of COVID-19 pandemic is considered. (2) In order to restrain the negative information of the public, policies and regulations information publicity with time delay and impulsive response will be proposed.
